# Factors associated with catastrophic health expenditure in sub-Saharan Africa: A systematic review

**DOI:** 10.1371/journal.pone.0276266

**Published:** 2022-10-20

**Authors:** Paul Eze, Lucky Osaheni Lawani, Ujunwa Justina Agu, Linda Uzo Amara, Cassandra Anurika Okorie, Yubraj Acharya

**Affiliations:** 1 Department of Health Policy and Administration, Pennsylvania State University, University Park, PA, United States of America; 2 Institute of Health Policy, Management & Evaluation, University of Toronto, Toronto, Canada; 3 Department of Community Medicine, Enugu State University Teaching Hospital, Parklane, Enugu, Nigeria; 4 Department of Community Medicine, Ebonyi State University Teaching Hospital, Abakaliki, Ebonyi State, Nigeria; Flinders University, AUSTRALIA

## Abstract

**Objective:**

A non-negligible proportion of sub-Saharan African (SSA) households experience catastrophic costs accessing healthcare. This study aimed to systematically review the existing evidence to identify factors associated with catastrophic health expenditure (CHE) incidence in the region.

**Methods:**

We searched PubMed, CINAHL, Scopus, CNKI, Africa Journal Online, SciELO, PsycINFO, and Web of Science, and supplemented these with search of grey literature, pre-publication server deposits, Google Scholar®, and citation tracking of included studies. We assessed methodological quality of included studies using the Appraisal tool for Cross-Sectional Studies for quantitative studies and the Critical Appraisal Skills Programme checklist for qualitative studies; and synthesized study findings according to the guidelines of the Economic and Social Research Council.

**Results:**

We identified 82 quantitative, 3 qualitative, and 4 mixed-methods studies involving 3,112,322 individuals in 650,297 households in 29 SSA countries. Overall, we identified 29 population-level and 38 disease-specific factors associated with CHE incidence in the region. Significant population-level CHE-associated factors were rural residence, poor socioeconomic status, absent health insurance, large household size, unemployed household head, advanced age (elderly), hospitalization, chronic illness, utilization of specialist healthcare, and utilization of private healthcare providers. Significant distinct disease-specific factors were disability in a household member for NCDs; severe malaria, blood transfusion, neonatal intensive care, and distant facilities for maternal and child health services; emergency surgery for surgery/trauma patients; and low CD4-count, HIV and TB co-infection, and extra-pulmonary TB for HIV/TB patients.

**Conclusions:**

Multiple household and health system level factors need to be addressed to improve financial risk protection and healthcare access and utilization in SSA.

**Protocol registration:**

PROSPERO CRD42021274830

## Introduction

Over 930 million people globally suffered undue financial hardship while obtaining healthcare and about 100 million people were forced into poverty yearly from out-of-pocket (OOP) health expenses in 2019 [1]. As the predominant healthcare financing system in sub-Saharan Africa (SSA), OOP payments have hindered the region’s drive towards universal health coverage (UHC) [2]. Besides, OOP healthcare financing is inefficient and highly inequitable, further impoverishing the poorest households in the region [2, 3].

Catastrophic health expenditure (CHE)–defined as OOP payment above an estimated threshold share of total household expenditure at which the household is forced to sacrifice other basic needs, sell assets, incur debts, or be impoverished [4]–engenders a vicious cycle of poverty for some households that choose to seek services and leads to more illnesses for those who cannot afford OOP costs [5]. Improving financial protection to minimize the extent to which households incur CHE and are pushed into poverty due to high medical spending has received substantial attention [1, 4, 6]. To this end, the United Nations in 2015 included CHE incidence as a key indicator to track progress towards UHC (SDG 3.8.2) [1, 4, 6]. Reducing CHE incidence is one of the key objectives of the global, regional, and national health policy drive towards UHC and human development [1, 5].

Our previous study had demonstrated that a non-negligible proportion of households annually experience CHE in SSA (16.5% at the 10% total household expenditure threshold and 8.8% at the non-food expenditure threshold) [7]. There is, however, a wide demand for a better understanding of the factors associated with catastrophic OOP expenditure in the region to fine-tune interventions to adequately protect households [5]. Hence, this study aims to systematically review the literature to identify the patients, household, and health system level factors associated with CHE incidence in SSA countries. For a comprehensive review, we sought both quantitative and qualitative studies, as qualitative studies may identify key themes not found, described, or discussed in quantitative studies [8, 9]. Our findings could help identify at-risk populations for community-wide and/or vertical disease-specific interventions.

## Methods

The protocol for this systematic review was registered on PROSPERO: CRD42021274830; and the findings reported according to the Preferred Reporting Items for Systematic Reviews and Meta-Analyses (PRISMA) guidelines [10].

### Search strategy

We searched PubMed, CINAHL, CNKI, AJOL, African Index Medicus, PsycINFO, SciELO, Scopus, and Web of Science for studies published from 01 January 2000 to 31 December 2021 conducted in any of the 48 World Bank-defined SSA countries. Two authors (PE and LOL) independently searched the literature in February 2022 using search terms covering catastrophic health expenditure, financial catastrophe, risk factors, “factors associated with”, and sub-Sahara Africa–**S1 Table**. Boolean operators “AND” and “OR” were used to broaden the search. We also searched grey literature websites: New York Academy of Medicine Grey Literature and Open Grey; pre-publication server deposits: medRxIV and PrePubMed; Google Scholar®; and tracked references of included studies for relevant articles. We considered studies published in any of the six African Union languages: Arabic, English, French, Kiswahili, Portuguese, and Spanish; and translated non-English publications using a translation service. We underwent a moderation exercise to ensure uniformity; screened abstracts according to prior eligibility criteria (**S2 Table**); retrieved full texts for eligible studies; and resolved discrepancies by discussion. We used Mendeley Desktop® to identify and remove duplicates.

### Data extraction

At least two authors (PE, LOL, LUA, CAO, and UJA) independently extracted data from included studies using a template. We extracted the following data from each included study: authors names, publication status, study setting, publication year, study design, data source and authors’ description of the data representativeness, study period, sampling method, sample size (in households), and factors associated with CHE. We extracted reported adjusted odds ratio with the confidence interval at 5.0% statistical significance for each CHE-associated factor. Where two or more studies used the same secondary data to identify CHE-associated factors, we first assessed both studies for unique factors, but if similar factors were evaluated, we then considered the peer-review status of the studies; prioritizing peer-reviewed studies over non-peer-reviewed studies. Where a study described CHE-associated factor using more than one CHE definition, we extracted data for both definitions {10% total household expenditure (THE) and 40% non-food expenditure (NFE)}. For qualitative studies; we manually extracted all text under the headings ‘results/conclusions’. We cross-checked all extracted data for discrepancies which were resolved through discussion.

### Risk of bias assessment

At least two authors (PE, CAO, LUA, UJA, and LOL) independently assessed the quality of included quantitative studies using the Appraisal tool for Cross-Sectional Studies (AXIS tool) [11], and the Critical Appraisal Skills Programme (CASP) checklist for qualitative studies [12]. We resolved discrepancies in quality assessment scores by discussion until 100% agreement. We categorized the articles’ quality into high (studies met ≥ 70% of the quality criteria), moderate (between 40% and 69% of the quality criteria), and low (< 40% of the quality criteria). We used Microsoft Excel® to organize extracted data.

### Data analysis

We first summarized the included studies descriptively. To synthesize the evidence, we performed meta-analysis and narrative synthesis following the Cochrane Handbook for Systematic Reviews of Interventions and the Economic and Social Research Council (ESRC) Methods Programme [9, 13] guidelines. We pooled studies reporting quantitative estimates (odds ratios) from regression or matching analysis for CHE-associated factors in a random-effects meta-analysis to obtain pooled effect estimates. Random effects meta-analysis allows for differences in the treatment effect from study to study because of real differences in the treatment effect in each study as well as sampling variability [14]. Analyses were conducted using Stata version 16.1 (STATA Corp, College Station, TX). Where meta-analysis was not possible due to difference in the definition of CHE-associated factors, we analyzed the reported quantitative estimates narratively.

For qualitative data, we independently performed line-by-line coding of text to group similar concepts and developed new codes when necessary. We organized free codes into descriptive major themes and sub-themes using an inductive approach as detailed by Thomas and Harden [15]. Each reviewer first did this independently and then as a group. Through discussion more abstract or analytical themes emerged and we resolved discrepancies between reviewers through discussion and consensus was achieved on all occasions. Finally, we globally assessed findings from both quantitative studies including meta-analysis for each CHE-associated factor–based of breadth of evaluation in included studies, consistency of an effect on CHE incidence, and methodological quality of included studies evaluating this factor–and when available, triangulated these with the participants’ lived experiences reported in qualitative studies to categorize each CHE-associated factor as either *significant* or *marginal*. We categorized a factor as “significant” if it was widely evaluated factors that consistently diminished or exaggerated the likelihood of CHE incidence. Otherwise, we categorized such factor as “marginal”.

### Deviations from study protocol

The original protocol was for a quantitative study. We decided to include qualitative studies to enrich our understanding of the key drivers of CHE based on individuals’ lived experiences, which population-based quantitative studies do not cover.

## Results

### Study characteristics

**Fig 1:**
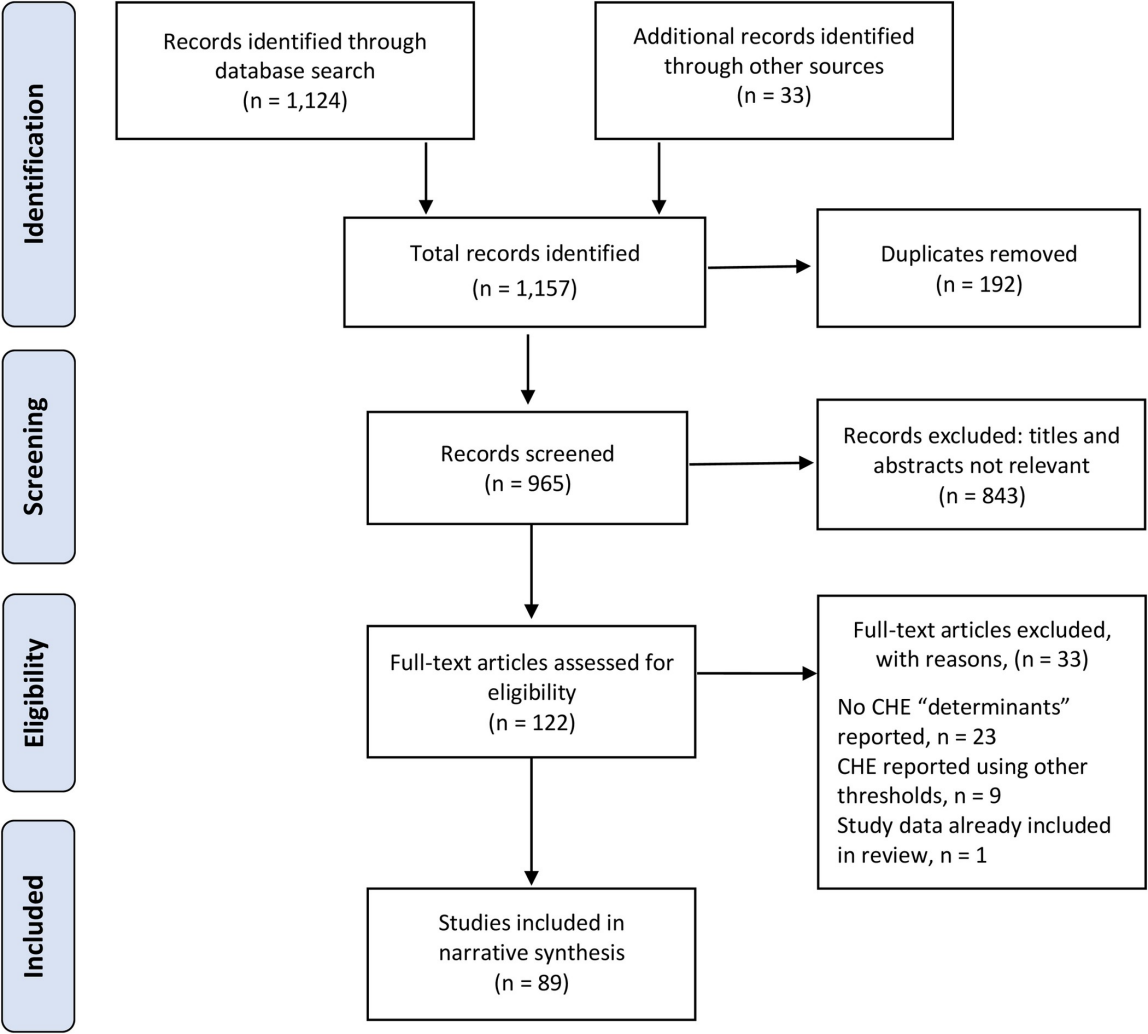
PRISMA flow diagram. PRISMA: Preferred Reporting Items for Systematic Reviews and Meta-Analyses; CHE: Catastrophic health expenditures.

**Fig 2:**
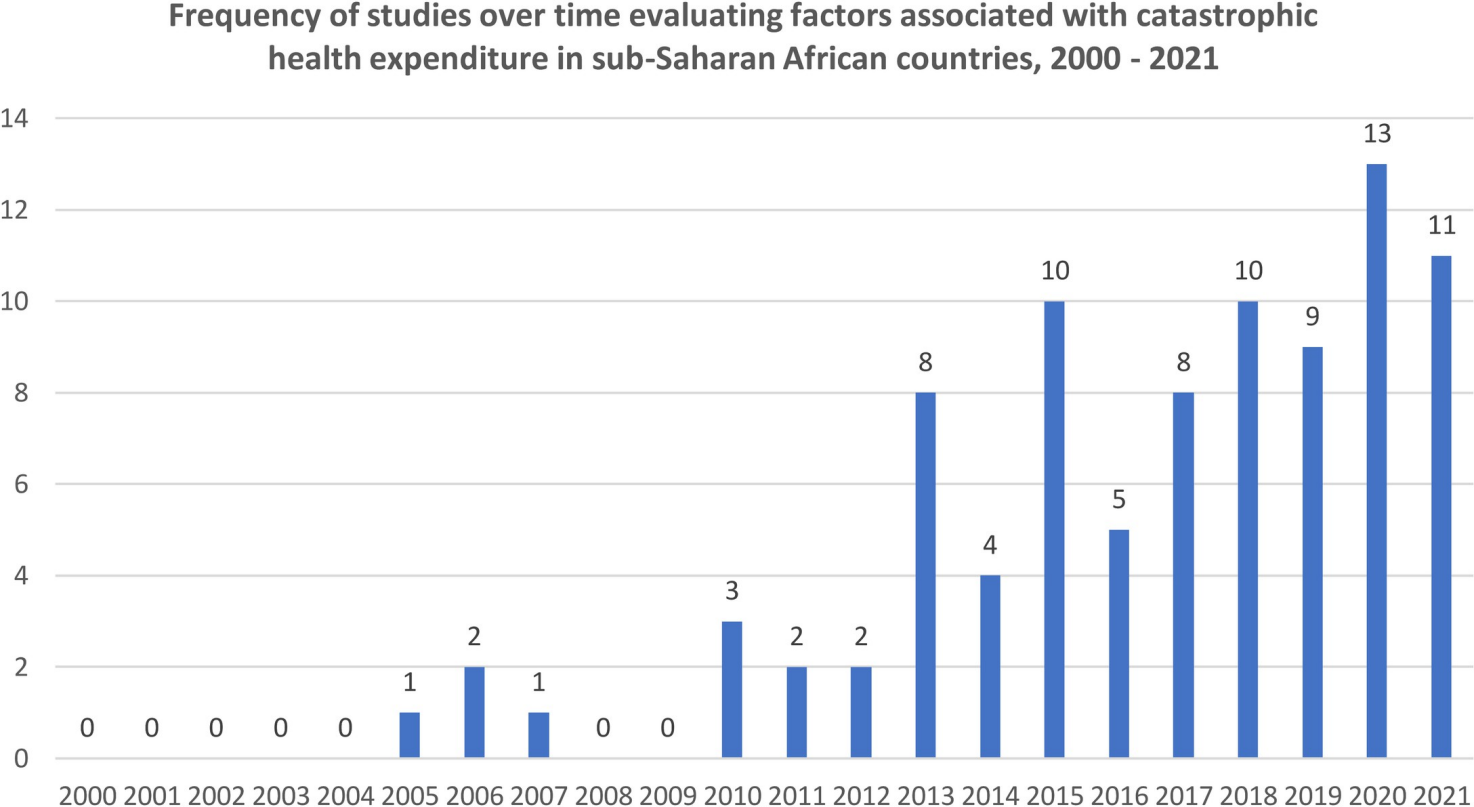
Frequency of included studies over time in sub-Saharan Africa, 2000–2021.

We identified 965 unique articles published between 2000 and 2021 (**Fig 1**). Of these articles, 122 full-text articles were screened for eligibility and 89 studies met inclusion criteria for this review [16–104] **(Table 1**). Included studies were 80 peer-reviewed publications, four working papers, and five dissertations, and covered 3,112,322 individuals in 650,297 households in 29 SSA countries. Included articles were published between 2005 to 2021 (**Fig 2**); were predominantly English-language articles (n = 85; 95.5%); mostly used nationally-representative samples (n = 48; 53.9%); and mostly estimated CHE incidence using ‘non-food expenditure’ definition (n = 53; 59.6%)–**Table 2**.

**Table 1:** Characteristics of included studies.

Study, Publication status	Study location (Country)	Study design	Data source, Study period (year)	Sample size (households)	CHE definition	Study health area	Study quality
Adeniji & Lawanson 2018 [16] *Published*	Nigeria	Cross-sectional study	Harmonized Nigeria Living Standard Survey (HNLSS), 2009/2010	38,700	40% NFE	General health care	Moderate
Adisa 2015 [17] *Published*	Nigeria	Cross-sectional study	Nigeria General Household & Population Survey (NGHPS), 2010	1,176	10% THE	General health care	High
Aidam et al. 2016 [18] *Published*	Ghana	Cross-sectional study	Primary data from household survey in Ga South Municipality, Ghana, 2013	117	40% NFE	General health care	Moderate
Akazili 2010 [19] *PhD thesis*	Ghana	Cross-sectional study	Ghana Living Standard Survey (GLSS), 2005	8,687	10% THE 40% NFE	General health care	High
Akinkugbe et al. 2012 [20] *Published*	Botswana Lesotho	Cross-sectional study	Botswana Household and Expenditure Survey (HIES), 2002/2003 and Lesotho Household budget Survey, 2002/2003	6,053 (BWA) 6,882 (LSO)	40% NFE	General health care	High
Aregbesola & Khan 2018 [21] *Published*	Nigeria	Cross-sectional study	Nigeria Harmonised Living Standard Survey (HNLSS), 2009/2010	38,700 ^a^	10% THE 40% NFE	General health care	High
Arsenault et al. 2013 [22] Published	Mali	Case-control study	Primary data from case–control study in Kayes region, Mali, 2008–2011	484	10% THE	Reproductive health (RH) services	High
Aryeetey et al. 2016 [23] *Published*	Ghana	Cross-sectional study	Primary data from household survey in Eastern and Central regions of Ghana, 2009	3,300	40% NFE	General health care	High
Assebe et al. 2020 [24] *Published*	Ethiopia	Cross-sectional study	Ethiopia Health Account (EHA); and health facility-based survey, 2016/2017	1,006 (HIV) 787 (TB)	10% THE	HIV/AIDS & Tuberculosis	High
Atake & Amendah 2018 [25] *Published*	Togo	Cross-sectional study	Primary data from household survey in Lomé, Togo, 2016	1,180	40% NFE	General health care	High
Attia-Konan et al. 2020 [26] *Published*	Cote d’Ivoire	Cross-sectional study	Cote d’Ivoire National household living standards survey, 2015	12,899	40% NFE	General health care	High
Babikir et al. 2018 [27] *Published*	South Africa	Panel survey	National Income Dynamics Study (NIDS), 2013	10,236	40% NFE	General health care	High
Barasa et al. 2017 [28] *Published*	Kenya	Cross-sectional study	Kenya Household Expenditure and Utilization Survey, 2013	33,675	40% NFE	General health care	High
Beauliere et al. 2010 [29] *Published*	Cote d’Ivoire	Cross-sectional study	Primary data from cross-sectional survey of 18 hospitals in Abidjan, Cote d’Ivoire, 2007	1,190	40% NFE	HIV/AIDS	High
Borde et al. 2020 [30] *Published*	Ethiopia	Cohort study	Primary data from cohort study in 3 kebeles in Wonago district, southern Ethiopia, 2017	794	10% THE 40% NFE	Reproductive health (RH) service	High
Botman et al. 2021 [31] Published	Tanzania	Mixed method (Survey & Observation)	Cross-sectional survey and observation of patients in a regional referral hospital in the Manyara, Tanzania, 2017	67	10% THE	Trauma (Burns patients)	High
Bousmah et al. 2021 [32] *Published*	Cameroun	Cross-sectional study	Primary data from two cross-sectional surveys in the HIV ART clinics in six regions in Cameroun, 2006/2007 and 2014	5,281	40% NFE	HIV/AIDS	High
Boyer et al. 2011 [33] *Published*	Cameroun	Cross-sectional study	Primary data from cross-sectional surveys in 27 hospitals in Cameroun, 2006–2007	3,151 ^a^	10% THE	HIV/AIDS	High
Brinda et al. 2014 [34] *Published*	Tanzania	Cross-sectional study	Tanzania National Panel Survey (TZNPS), 2008/2009	3,265	40% NFE	General health care	High
Buiguit et al. 2015 [35] *Published*	Kenya	Cross-sectional study	Indicator Development for Surveillance of Urban Emergencies Project, 2011	8,171	10% THE	General health care	High
Chabrol et al. 2019 [36] *Published*	Cameroun	Qualitative	Interviews with affected patients in reference hospitals in Yaoundé, Cameroon, 2014	12	10% THE	HBV and HCV	High
Chukwu et al. 2017 [37] *Published*	Nigeria	Cross-sectional study	Primary data from multi-hospital survey in four states in Nigeria, 2015	92	10% THE	Buruli ulcer (NTD)	High
Cleary et al. 2013 [38] *Published*	South Africa	Cross-sectional study	Primary data from household survey in four provinces (Western Cape, Guateng, Mpumalanga, & KwaZulu Nata) in South Africa, 2011	1,267 (HIV) 1,231 (RHS) 1,229 (TB)	10% THE	HIV/AIDS, RH services, & Tuberculosis	High
Counts & Skordis-Worrall 2016 [39] *Published*	Tanzania	Panel survey	Kagera Health and Development Surveys, 1991–2010	900	40% NFE	General health care	High
Dhufera et al. 2022 [40] *Published*	Ethiopia	Cross-sectional study	Primary data from trauma units of multiple hospitals in Addis Ababa, Ethiopia, 2019	452	40% NFE	Trauma	High
Doamba et al. 2013 [41] *Published* {FRENCH}	Burkina Faso	Cross-sectional study	Burkina Faso Enquête Intégrale sur les Conditions de Vie des Ménages (EICVM), 2009	8,404	40% NFE	General health care	Moderate
Dyer et al. 2013 [42] *Published*	South Africa	Prospective cohort study	Primary data from cross-sectional hospital survey, Cape Town, South Africa 2009 to 2011	148	40% NFE	Reproductive Health (RH) services	High
Ebaidalla & Ali 2019 [43] *Published*	Sudan	Cross-sectional study	Sudan National Baseline Households Survey (NBHS), 2009.	7,913 ^a^	40% NFE	General health care	High
Ebaidalla 2021 [44] *Published*	Sudan	Cross-sectional study	Sudan National Baseline Household Survey (NBHS), 2009 and 2014	7,913 (2009) 11,953 (2014)	10% THE	General health care	Moderate
Edoka et al. 2017 [45] *Published*	Sierra Leone	Cross-sectional study	Sierra Leone integrated household survey (SLIHS), 2003 and 2011	6,800 (2003) 3,700 (2011)	10% THE	General health care	High
Ekman 2007 [46] *Published*	Zambia	Cross-sectional study	Zambian Living Conditions Monitoring Survey II (LCMS II), 1998	16,000	10% THE	General health care	High
Fink et al. 2013 [47] *Published*	Burkina Faso	Pre-intervention baseline survey	Nouna Health and Demographic Surveillance System Survey, 2003	983	10% THE	General health care	High
Hailemichael et al. 2019 [49] *Published*	Ethiopia	Case-control study	Primary data from population-based, cross-sectional study in Sodo district of the Southern Nations, Nationalities and Peoples’ Regional State, Ethiopia, 2015	257	10% THE	Chronic NCDs	High
Hailemichael et al. 2019 [48] *Published*	Ethiopia	Case-control study	Primary data from a population-based cross-sectional household survey in Sodo district in southern Ethiopia, 2015	579	40% NFE	Chronic NCDs	High
Hilaire 2018 [50] *Working Paper*	Benin	Cross-sectional survey	Benin Integrated Modular Survey on Living Condition of Households, 2009	15,411	10% THE	General health care	High
Ibukun & Adebayo 2021 [51] *Published*	Nigeria	Mixed method (Survey & Interviews)	Cross-sectional survey and interviews of hospital patients in Nigeria, 2019	1,320	40% NFE	Chronic NCDs	High
Ibukun & Komolafe 2018 [52] *Published*	Nigeria	Cross-sectional survey	Nigeria General Household Survey Panel (GHS), 2015/2016	4,581	40% NFE	General health care	Moderate
Ilesanmi et al. 2014 [53] *Published*	Nigeria	Cross-sectional survey	Primary data from household survey in Oyo State, SW Nigeria, 2012	714	40% NFE	General health care	High
Ilunga-Ilunga et al. 2015 [54] *Published*	Congo, DR	Cross-sectional survey	Primary data from multi-hospital survey in Kinshasa, Congo DR, 2012	1,350	10% THE 40% NFE	Malaria	High
Janssen et al. 2016 [55] *Published*	Nigeria	Cross-sectional study	Primary data from household survey in rural Kwara State, Nigeria, 2009	1,450	40% NFE	General health care	High
Kaonga et al. 2019 [56] *Published*	Zambia	Cross-sectional study	Zambian Household Health Expenditure and Utilisation Survey, 2014	12,000	10% THE	General health care	High
Kasahun et al. 2020 [57] *Published*	Ethiopia	Cross-sectional survey	Primary data from cross-sectional from multiple hospitals in Addis Ababa, Ethiopia, 2018	352	10% THE	Chronic NCDs	High
Khatry et al. 2013 [58] *Published* {FRENCH}	Mauritania	Cross-sectional study	Mauritania Enquête Permanente sur les Conditions de Vie des ménages (EPCV), 2008	13,705	40% NFE	General health care	Moderate
Kihaule 2015 [59] *Published*	Tanzania	Cross-sectional survey	Tanzania Demographic and Health Survey, 2009	10,300	40% NFE	General health care	Moderate
Kimani et al. 2016 [60] *Published*	Kenya	Cross-sectional study	Kenya Household Expenditure Utilization Survey (KHHEUS), 2007	8,844	10% THE 40% NFE	General health care	Low
Kirubi et al. 2021 [61] *Published*	Kenya	Cross-sectional survey	Kenya National Tuberculosis Programme Patient Cost Survey, 2017	1,071	10% THE	Tuberculosis	High
Kusi et al. 2015 [62] *Published*	Ghana	Cross-sectional study	Primary data from cross-sectional household survey in Kwaebibirem, Asutifi, and Savelugu-Nanton districts, Ghana, 2011	2,430	40% NFE	General health care	High
Kwesiga et al. 2020 [63] *Published*	Uganda	Cross-sectional study	Uganda National Household Survey (NHS), 2005/2006, 2009/2010, 2012/2013, 2016/2017	7,400 (2005) 6,887 (2009) 7,500 (2012) 17,320 (2016)	10% THE	General health care	High
Lamiraud et al 2005 [64] *Working Paper*	South Africa	Cross-sectional study	World Health Survey, 2002	2,602	40% NFE	General health care	Moderate
Lu et al. 2012 [65] *Published*	Rwanda	Cross-sectional study	Rwanda Integrated Living Conditions Survey, 2000	6,408	40% NFE	General health care	Moderate
Lu et al. 2017 [66] *Published*	Rwanda	Cross-sectional study	Rwanda Integrated Living Conditions Survey, 2005 & 2010	6,900 (2005) 14,308 (2010)	40% NFE	General health care	High
Masiye et al. 2016 [67] *Published*	Zambia	Cross-sectional study	Zambia Household Health Expenditure & Utilization Survey (ZHHEUS), 2014	11,847	10% THE	General health care	High
Mulaga et al. 2021 [68] *Published*	Malawi	Cross-sectional study	Malawi Integrated Household Survey (IHS4), 2016/2017	12,447	10% THE 40% NFE	General health care	High
Muttamba et al. 2020 [69] *Published*	Uganda	Cross-sectional study	Primary data from cross-sectional survey in 67 TB diagnostic and treatment units in Uganda, 2017	1,178	10% THE	Tuberculosis	High
Mutyambizi et al. 2019 [70] Published	South Africa	Cross-sectional	Primary data from cross-sectional survey at two public hospitals in Tshwane, Gauteng State, South Africa, 2017	395	40% NFE	Chronic NCDs	High
Mwai & Muriithi 2016 [71] *Published*	Kenya	Cross-sectional study	Kenya Household Health Expenditure & Utilization Survey (KHHEUS), 2007	8,453 ^a^	40% NFE	General health care	Low
Negin et al. 2016 [72] *Published*	South Africa	Cross-sectional study	Study on global AGEing and adult health (SAGE) South Africa Wave 1, 2007/2008.	2,969	40% NFE	General health care	High
Ngcamphalala 2015 [73] *MPH thesis*	Eswatini (Swaziland)	Cross-sectional study	Swaziland Household Income and Expenditure Survey (SHIES), 2009/2010	3,167	10% THE	General health care	Moderate
Nguyen et al. 2011 [74] *Published*	Ghana	Cross-sectional study	Primary data from household survey in Nkoranza and Offinso districts, Ghana, 2007	2,500	10% THE	General health care	High
Njagi et al. 2020 [75] *Published*	Kenya	Cross-sectional study	Kenya Household Health Expenditure & Utilisation Survey, 2007 and 2013	3,728 (2007) 16,526 (2013)	40% NFE	General health care	High
Njuguna et al. 2017 [76] *Published*	Kenya	Cross-sectional study	Kenya Household Health Utilization & Expenditure Survey (KHHUES), 2013	33,675 ^a^	40% NFE	General health care	Low
Ntambue et al. 2019 [77] *Published*	Congo, DR	Mixed method (Survey & Interviews)	Cross-sectional survey and interviews of hospital patients in Nigeria, 2015	1,627	40% NFE	Reproductive Health (RH) services	High
Nundoochan et al. 2019 [78] *Published*	Mauritius	Cross-sectional study	Mauritius Household Budget Surveys, 2001/2002, 2006/2007, and 2012	6,720 (2001) 6,720 (2006) 6,720 (2012)	10% THE 40% NFE	General health care	High
Nwanna-Nzewunwa et al. 2021 [79] *Published*	Uganda	Mixed method (Prospective cohort and Qualitative)	Survey and interviews of affected patients at Soroti Regional Referral Hospital Uganda, 2018/2019	546	10% THE	Surgery	High
Nyankangi et al. 2020 [80] *MSc Thesis*	Kenya	Cross-sectional study	Kenya Household Health Utilization & Expenditure Survey (KHHUES), 2018	37,500	40% NFE	Chronic NCD	High
Obembe & Fonn 2020 [81] *Published*	Nigeria	Qualitative study	Interviews with patients and family members liable for paying for surgery in Ibadan, Nigeria, 2017	31	10% THE	Emergency surgery	High
Obembe et al. 2021 [82] *Published*	Nigeria	Cross-sectional study	Primary data from cross-sectional household survey in Ibadan, Oyo State, Nigeria, 2017	450	10% THE	Emergency surgery	High
Ogaji & Adesina 2018 [83] *Published*	Nigeria	Cross-sectional study	Primary data from cross-sectional household survey in Yenagoa, Bayelsa St, Nigeria, 2012	525	10% THE	General health care	Moderate
Okoroh et al. 2020 [84] *Published*	Ghana	Cross-sectional study	Primary data from cross-sectional survey at a regional referral hospital, Accra, Ghana, 2017	196	40% NFE	Surgery	High
Olatunya et al. 2015 [85] *Published*	Nigeria	Cross-sectional study	Primary data from cross-sectional survey at a regional referral hospital Ado Ekiti, Ekiti State, 2014	111	10% THE	Chronic NCD	High
Onah & Govender 2014 [86] *Published*	Nigeria	Cross-sectional study	Primary data from cross-sectional survey in Nsukka LGA, Nigeria, 2012	411	10% THE	General	High
Onarheim et al. 2018 [87] *Published*	Ethiopia	Qualitative study	Interviews and focus group discussions with caretakers Ethiopia, 2015	41 interviews and 7 FGDs	10% THE	Newborn	High
Owusu-Sekyere 2015 [88] *MPhil thesis*	Ghana	Cross-sectional study	Ghana Living Standards Survey (GLSS 6), 2012	16,772	40% NFE	General	Moderate
Petitfour et al. 2021 [89] *Published*	Burkina Faso	Cross-sectional study	Primary data from cross-sectional survey at the sole referral hospital in Ouagadougou, Burkina Faso, 2015	1,323	10% THE	Trauma	High
Rickard et al. 2017 [90] *Published*	Rwanda	Cross-sectional study	Primary data from cross-sectional survey at a regional referral hospital, Kigali Rwanda, 2014/2015	245	40% NFE	Surgery	High
Saksena et al. 2010 [91] *Working paper*	Burkina Faso Chad Congo, Rep Cote d’Ivoire Ethiopia Ghana Kenya Malawi Mali Mauritania Mauritius Namibia Swaziland Zambia Zimbabwe	Cross-sectional study	WHO World Health Survey, 2002–2003	4,948 (BFA) 4,875 (TCD) 3,070 (COG) 3,245 (CIV) 5,090 (ETH) 4,165 (GHA) 4,640 (KEN) 5,551 (MWI) 5,209 (MLI) 3,907 (MRT) 3,958 (MUS) 4,379 (NAM) 3,121 (SWZ) 6,165 (ZMB) 4,264 (ZWE)	40% NFE	General health care	High
Salari et al. 2019 [92] *Published*	Kenya	Cross-sectional study	Kenya Household Health Utilization & Expenditure Survey (KHHUES), 2018	37,500 ^a^	10% THE 40% NFE	General health care	High
Sanoussi & Ameteglo 2019 [93] {FRENCH} *Working paper*	Togo	Cross-sectional study	Togo Questionnaire of Basic Indicators of Well-being (QUIBB) survey, 2015	2,400	10% THE 40% NFE	General health care	Moderate
Sene & Cisse 2015 [94] *Published*	Senegal	Cross-sectional study	Senegal Poverty Monitoring Survey, 2011	5,953	10% THE	General health care	Moderate
Shikuro et al. 2020 [95] *Published*	Ethiopia	Cross-sectional study	Primary data from cross-sectional household survey, 2017	479	40% NFE	General health care	High
Shumet et al. 2021 [96] *Published*	Ethiopia	Cross-sectional study	Primary data from cross-sectional study in Mandura, Ethiopia, 2018	302	10% THE	Chronic NCDs	High
Sichone 2020 [97] *Graduate MSc thesis*	Zambia	Cross-sectional study	Zambia Household Health Expenditure & Utilisation Survey, 2014	2,164	10% THE	Malaria in children < 5 year of age	High
Sow et al. 2013 [98] {FRENCH} *Published*	Senegal	Cross-sectional study	Senegal Enquêtes de Suivi de la Pauvreté au Sénégal, 2011	18,000	40% NFE	General health care	Moderate
Su et al. 2006 [99] *Published*	Burkina Faso	Cross-sectional study	Nouna Health District Household Survey (NHDHS), 2000/2001	774	40% NFE	General health care	Moderate
Tolla et al. 2017 [100] *Published*	Ethiopia	Cross-sectional study	Primary data from cross-sectional survey of CVD patients in Addis Ababa, Ethiopia, 2015	589	10% THE	Chronic NCD	High
Tsega et al. 2021 [101] *Published*	Ethiopia	Cross-sectional study	Primary data from cross-sectional survey of CVD patients in Bahir Dar city, Ethiopia, 2019	422	40% NFE	Chronic NCD	High
Ukwaja et al. 2013 [102] *Published*	Nigeria	Cross-sectional study	Primary data from population-based household survey, 2011	452	40% NFE	Tuberculosis	High
Xu et al. 2006 [103] *Published*	Uganda	Cross-sectional study	Uganda Socio-economic Surveys (USS), 2000 and 2003	10,691 (2000) 9,710 (2003)	40% NFE	General health care	High
Zeng et al. 2018 [104] *Published*	Zimbabwe	Cross-sectional study	Zimbabwe National Statistics Agency Household Survey, 2016	7,135	10% THE	General health care	High

^**a**^ Samples were not included in the overall study participants total reported

**NCD**: Non-communicable disease, **NTD**: Neglected tropic disease, **RH**: Reproductive health, **TB**: Tuberculosis

**BFA**: Burkina Faso, **TCD**: Chad, **COG**: Congo, Republic, **CIV**: Cote d’Ivoire, **ETH**: Ethiopia, **GHA**: Ghana, **KEN**: Kenya, **MWI**: Malawi, **MLI**: Mali, **MRT**: Mauritania, **MUS**: Mauritius, **NAM**: Namibia, **SWZ**: Eswatini (Swaziland), **ZMB**: Zambia, and **ZWE**: Zimbabwe

**Table 2:** Summary characteristics of included studies.

Study characteristics	Frequency (%)
	N = 89
**Sub-Saharan Africa region**	
◦ Central SSA	6 (6.7%)
◦ East SSA	37 (41.6%)
◦ South SSA	20 (22.5%)
◦ West SSA	41 (46.1%)
**Top-five study countries**	
◦ Nigeria	14 (15.7%)
◦ Ethiopia	12 (13.5%)
◦ Kenya	10 (11.2%)
◦ Ghana	8 (9.0%)
◦ South Africa	6 (6.7%)
**Study population**	
◦ General (Entire community)	52 (58.4%)
◦ Patients with NCDs	10 (11.2%)
◦ Pregnant and nursing mothers and newborns	6 (6.7%)
◦ Infectious diseases (HIV, TB, HBV, and HCV)	10 (11.2%)
◦ Surgery and trauma	8 (9.0%)
◦ Malaria and neglected tropical diseases	4 (4.5%)
**Study language**	
◦ English	85 (95.5%)
◦ French	4 (4.5%)
**Definition of catastrophic health expenditure used**	
◦ 10% total household expenditure	36 (40.5%)
◦ 40% non-food expenditure	43 (48.3%)
◦ Both definitions	10 (11.2%)
**Study design**	
◦ Quantitative	82 (92.1%)
◦ Qualitative	3 (3.4%)
◦ Mixed methods	4 (4.5%)
**Data source**	
◦ Primary data	41 (46.1%)
◦ Secondary source	48 (53.9%)
**Publication status**	
◦ Peer-reviewed	80 (89.9%)
◦ Non-peer-reviewed	9 (10.1%)
**Study quality**	
◦ High quality	70 (78.6%)
◦ Moderate quality	16 (18.0%)
◦ Low quality	3 (3.4%)

Of the 89 included studies, 70 (78.6%) were rated as high quality, 16 (18.0%) as moderate quality, and the remaining 3 (3.6%) as low quality–**Table 1**. Of note, all included quantitative studies used sample frames that closely represented the target population (AXIS tool Item 5) and used selection procedures that likely selected samples representative of the underlying population (AXIS tool Item 6). Also, included qualitative studies used sampling techniques that ensured the identification and selection of individuals that recently suffered catastrophic health expenses.

### Catastrophic health expenditure-associated factors

Included studies involved 82 population-based studies reporting quantitative estimates, of which a total of 73 were included in the 71 different random-effects meta-analysis. Nine studies were included in narrative synthesis. Quantitative data from four mixed methods studies were also included in the narrative synthesis. Results from quantitative meta-analysis were reported in two broad categories: population-level factors and disease-specific factors (**Tables 3 and 4**). Seven studies reporting qualitative data (3 qualitative studies and 4 mixed-methods) met the inclusion criteria, all of which were included in thematic analysis (**Table 5**). Qualitative data revealed two main themes associated with households’ CHE incidence: low socioeconomic status and being uninsured (**Table 6**). We presented excerpts of supportive qualitative findings with the relevant quantitative findings and a thematic analysis map in **S1 Fig**.

**Table 3:** Socio-demographic factors associated with population-level catastrophic health expenditure in SSA countries.

	10% Total Household Expenditure			40% Non-Food Expenditure			Authors’ global assessment of factor’s weight
	No. of studies	Sample size	Pooled OR (95% CI)	No. of studies	Sample size	Pooled OR (95% CI)	
**Household characteristics**							
◦ Residence *(ref = urban)*	14	227,692	1.01 (0.93–1.09)	15	299,595	1.11 (0.93–1.36)	*Significant*
◦ Socioeconomic status *(ref = wealthiest)*	15	216,086	1.99 (1.32–2.98)	20	284,017	3.02 (2.23–4.08)	*Significant*
◦ Household size	10	160,933	1.07 (1.02–1.13)	18	258,456	1.06 (0.99–1.12)	*Significant*
◦ Insurance status *(ref = insured)*	9	123,203	1.69 (0.69–4.16)	14	242,511	1.16 (0.65–2.08)	*Significant*
◦ Social safety net (benefits, vouchers, etc.)	1	8,171	0.63 (0.52–0.79)	1	10,236	1.29 (1.14–1.44)	*Marginal*
◦ Marginalization status	0			1	33,675	1.38 (1.14–1.67)	*Marginal*
**Household head characteristics**							
◦ Sex *(ref = Male)*	14	219,721	1.03 (0.95–1.11)	17	290,879	1.04 (0.97–1.11)	*Marginal*
◦ Age *(ref = young adult*, *< 40 years)*	8	109,687	1.07 (1.00–1.15)	11	183,036	0.96 (0.83–1.11)	*Marginal*
◦ Marital status: widowed/divorced *(ref = married)*	6	116,802	0.97 (0.93–1.03)	8	169,958	1.00 (0.94–1.07)	*Marginal*
◦ Education *(ref = at least secondary educ*.*)*	12	199,103	1.13 (0.94–1.35)	16	257,656	1.13 (0.94–1.35)	*Marginal*
◦ Employment status *(ref = employed)*	13	220,874	1.19 (0.96–1.48)	10	212,289	1.16 (1.05–1.29)	*Significant*
**Household members**							
◦ Presence of children < 5 years old	9	148,188	1.07 (1.00–1.15)	10	147,023	0.96 (0.83–1.11)	*Marginal*
◦ Presence of women of reproductive age	1	525	0.19 (0.10–0.36)	0			*Marginal*
◦ Presence of elderly person	12	193,093	1.06 (1.03–1.08)	13	239,345	1.30 (1.15–1.47)	*Significant*
◦ Chronic illness in a household member	9	128,419	2.12 (1.76–2.55)	14	222,213	1.93 (1.62–2.31)	*Significant*
◦ Hospitalization of a household member	5	27,236	2.62 (0.93–7.42)	6	70,852	3.91 (2.07–7.35)	*Significant*
◦ Disability in a household member	1	1,176	0.84 (0.47–1.48)	4	36,687	1.10 (0.82–1.46)	*Marginal*
◦ Smoker *(ref = non-smoker)*	0			1	38,700	1.11 (1.10–1.12	*Marginal*
◦ Obesity/Overweight	0			1	4,842	1.02 (0.91–1.34)	*Marginal*
**Health system factors**							
◦ Health facility level *(ref = primary care)*	4	42,518	1.85 (1.18–2.91)	1	1,180	3.82 (1.36–19.72)	*Significant*
◦ Health facility type: private *(ref = public)*	9	94,514	1.18 (0.48–2.90)	6	74,981	1.08 (0.36–3.26)	*Significant*
◦ Health facility type: mission *(ref = public)*	4	35,785	2.17 (0.70–6.69)	1	12,447	2.28 (1.24–4.15)	*Marginal*
◦ Distance to health facility	5	54,694	1.01 (1.00–1.03)	4	32,344	1.01 (0.68–1.50)	*Marginal*
◦ Number of health facilities in county	0			1	33,675	1.00 (1.00–1.02)	*Marginal*
◦ First sought care from traditional healers	0			2	18,533	1.64 (0.42–6.44)	*Marginal*
**Other factors**							
◦ Violence against women	0			1	8,297	1.41 (1.05–1.91)	*Marginal*
◦ Owns house *(ref = rent/lease house)*	1	16,000	1.86 (1.17–2.97)	0			*Marginal*
◦ Regular use of mosquito bed nets	1	1,176	1.35 (0.83–2.20)	0			*Marginal*
◦ Owns business	1	8,171	1.02 (0.86–1.22)	0			*Marginal*

Abbreviations: **HF**: Health facility

**Table 4:** Socio-demographic factors associated with disease-specific catastrophic health expenditure in SSA countries.

	10% Total household expenditure			40% Non-food expenditure			Authors’ global assessment of factor’s weight
	No. of studies	Sample size	Pooled OR (95% CI)	No. of studies	Sample size	Pooled OR (95% CI)	
**Non-communicable diseases (NCDs)**							
◦ Residence *(ref = urban)*	3	1,250	1.29 (0.51–3.29)	4	46,924	1.00 (0.78–1.28)	*Significant*
◦ Socioeconomic status *(ref = wealthiest)*	5	8,803	4.72 (1.05–21.24)	4	39,821	1.33 (1.06–1.68)	*Significant*
◦ Household size	1	1,056	2.06 (0.75–5.60)	2	10,322	1.06 (0.92–1.21)	*Marginal*
◦ Insurance status *(ref = insured)*	0			3	47,243	1.01 (0.93–1.10)	*Significant*
◦ Household head sex *(ref = male)*	1	257	1.11 (0.48–3.33)	2	1,899	1.25 (1.10–1.43)	*Marginal*
◦ Patients’ sex *(ref = male)*	2	799	1.05 (0.44–2.53)	3	46,345	1.09 (0.88–1.36)	*Marginal*
◦ Patients’ marital status: widow/divorced *(ref = married)*	2	706	2.72 (0.88–8.38)	3	46,345	1.00 (0.89–1.12)	*Marginal*
◦ Patients’ education *(ref = at least secondary educ*.*)*	3	1,056	0.77 (0.35–1.69)	2	38,501	1.23 (0.65–2.33)	*Marginal*
◦ Patients’ employment status *(ref = employed)*	3	1,388	0.99 (0.50–1.96)	3	37,922	1.53 (1.01–2.34)	*Marginal*
◦ Presence of elderly persons in household	4	1,645	1.01 (0.99–1.03)	2	38,079	1.03 (1.02–1.04)	*Marginal*
◦ Presence of children in household	1	257	0.50 (0.20–1.60)	1	1,001	0.69 (0.45–1.05)	*Marginal*
◦ Chronic illness in a household member	1	257	2.10 (1.10–4.60)	3	46,502	1.26 (1.11–1.43)	*Significant*
◦ Disability in a household member	1	257	2.10 (1.10–4.60)	1	579	1.50 (1.00–2.70)	*Significant*
◦ Health facility type *(ref = public)*	2	993	5.42 (0.36–82.13)	2	1,742	1.00 (0.49–2.07)	*Marginal*
◦ Duration of NCDs diagnosis	1	589	0.99 (0.98–0.99)	0			*Marginal*
**Reproductive, maternal, newborn, & child Health**							
◦ Residence *(ref = urban)*	1	484	7.14 (2.51–20.41)	0			*Significant*
◦ Socioeconomic status *(ref = wealthiest)*	1	1,231	0.87 (0.71–1.07)	2	1,775	33.97 (1.70–67.74)	*Significant*
◦ Insurance status *(ref = insured)*	0			1	148	2.11 (0.92–4.80)	*Marginal*
◦ Mothers’ marital status *(ref = married)*	0			1	1,627	2.40 (1.50–3.50)	*Marginal*
◦ Mothers’ education *(ref = at least secondary educ)*	2	1,715	2.05 (0.49–8.49)	1	148	0.02 (0.01–0.03)	*Marginal*
◦ Household head employment status *(ref = employed)*	1	1,231	1.06 (0.87–1.29)	0			*Marginal*
◦ Distance to Health facility > 5km (ref < 5 km)	1	484	1.02 (0.43–2.41)	0			*Significant*
◦ Distance to Health facility > 40 km (ref < 5 km)	1	484	2.54 (1.22–5.30)	0			*Significant*
◦ Blood transfusion	1	484	2.78 (1.47–5.25)	0			*Significant*
◦ Complicated vaginal delivery *(ref = UVD)*	0			1	1,627	1.80 (1.40–2.40)	*Marginal*
◦ Caesarean delivery *(ref = UVD)*	0			1	1,627	5.00 (3.90–6.30)	*Marginal*
◦ Delivery at unplanned facility	0			1	1,627	1.30 (1.10–1.70)	*Marginal*
◦ Referral status *(ref = not referred)*	0			1	1,627	2.80 (2.20–3.60)	*Marginal*
◦ Neonatal Intensive care unit admission	1	794	2.56 (1.02–6.44)	1	1,627	2.40 (1.90–3.10)	*Significant*
**Surgery and trauma care**							
◦ Residence *(ref = urban)*	1	450	1.03 (0.17–5.56)	1	452	2.95 (1.80–4.80)	*Significant*
◦ Socioeconomic status *(ref = wealthiest)*	1	450	31.3 (4.42–221.86)	1	452	2.23 (1.13–4.90)	*Significant*
◦ Insurance status *(ref = insured)*	1	450	5.88 (4.55–333.33)	2	648	9.54 (3.23–28.16)	*Significant*
◦ Sex of household head *(ref = male)*	2	1,773	1.00 (0.98–1.01)	0			*Marginal*
◦ Age of household head *(ref = > 40 years)*	2	1,773	1.00 (0.96–1.04)	0			*Marginal*
◦ Marital status of HH *(ref = married)*	1	450	1.59 (0.07–33.33)	1	NS	NS	*Marginal*
◦ Education *(ref = at least secondary educ*.*)*	2	1,773	0.98 (0.96–1.02)	1	NS	NS	*Marginal*
◦ Employment status *(ref = employed)*	1	450	3.85 (0.38–43.48)	1	NS	NS	*Marginal*
◦ Presence of elderly persons in household	0			1	NS	NS	*Marginal*
◦ Health facility type *(ref = public)*	1	450	0.73 (0.24–2.22)	1	452	6.50 (2.60–15.80)	*Marginal*
◦ Health facility level *(ref = primary care)*	1	450	3.19 (1.00–10.16)	0			*Marginal*
◦ Hospitalization	0			1	452	10.80 (5.40–24.80)	*Significant*
◦ Intensive care unit (ICU) admission	0			1	280	1.81 (0.73–1.51)	*Marginal*
◦ Length of hospital stay	0			1	280	1.04 (0.99–1.08)	*Marginal*
◦ Emergency/unplanned surgery	0			1	280	5.76 (2.14–15.54)	*Significant*
◦ Religion *(ref = Christian)*	1	450	2.59 (0.54–12.42)	0			*Marginal*
**HIV/AIDS, TB, HBV, and HCV**							
◦ Residence *(ref = urban)*	1	3,151	1.75 (1.36–2.26)	3	41,659	0.83 (0.32–2.14)	*Marginal*
◦ Socio-economic status *(ref = wealthiest)*	4	6,602	1.79 (0.19–17.03)	4	42,111	1.57 (0.40–6.25)	*Marginal*
◦ Household size	0			1	1,190	0.73 (0.66–0.81)	*Marginal*
◦ Insurance status *(ref = insured)*	1	1,006	2.70 (1.10–6.70)	3	41,659	0.96 (0.44–2.12)	*Marginal*
◦ Social safety net (benefits, vouchers, etc.)	1	1,267	1.00 (1.00–1.00)	0			*Marginal*
◦ Sex of the patient *(ref = male)*	1	3,151	0.97 (0.77–1.23)	4	42,111	1.04 (0.58–1.88)	*Marginal*
◦ Married status of patient	1	3,151	0.53 (0.45–0.64)	0			*Marginal*
◦ Age of head of household	0			1	2,969	1.00 (0.01–1.10)	*Marginal*
◦ Education *(ref = at least secondary educ*.*)*	0			3	4,611	1.74 (0.62–4.85)	*Marginal*
◦ Employment status *(ref = employed)*	1	1,267	1.06 (0.87–1.29)	2	38,690	1.34 (1.12–1.60)	*Marginal*
◦ Elderly household member	0			1	452	3.90 (2.00–7.80)	*Marginal*
◦ Hospitalization	1	1,006	30.60 (4.80–199.80)	0			*Marginal*
◦ Health facility type *(ref = public)*	1	1,006	2.60 (1.50–4.30)	1	452	2.90 (1.50–8.90)	*Marginal*
◦ Decentralization of care	1	3,151	0.53 (0.42–0.67)	0			*Marginal*
◦ CD4 count *(ref = ≥350)*	1	3,151	1.00 (1.00–1.00)	1	1,190	1.04 (0.51–2.11)	*Significant*
◦ HIV and TB co-infection	2	2,184	1.72 (0.55–5.36)	1	452	3.10 (1.70–5.60)	*Significant*
◦ Extra-pulmonary tuberculosis	1	1,006	2.60 (1.80–4.00)	0			*Significant*
◦ Duration on anti-retroviral therapy	1	2,412	1.00 (0.99–1.01)	1	1,190	0.97 (0.94–0.99)	*Marginal*
◦ Delay in diagnosis	1	1,178	1.10 (0.70–1.80)	0			*Marginal*
**Malaria**							
◦ Residence *(ref = urban)*	2	3,514	0.92 (0.78–1.08)	0			*Marginal*
◦ Socioeconomic status *(ref = wealthiest)*	2	3,514	3.98 (0.15–108.22)	1	1,350	13.00 (7.90–21.20)	*Significant*
◦ Sex of household head *(ref = male)*	1	2,164	1.08 (0.92–1.26)	1	1,350	2.90 (1.20–6.90)	*Marginal*
◦ Age of household head	1	2,164	0.98 (0.93–1.05)	0			*Marginal*
◦ Education *(ref = at least secondary educ*.*)*	1	2,164	0.96 (0.68–1.37)	0			*Marginal*
◦ Employment status *(ref = employed)*	1	2,164	1.05 (0.86–1.29)	0			*Marginal*
◦ Health facility type *(ref = public)*	2	3,514	2.34 (0.99–5.51)	1	1,350	3.70 (2.50–5.50)	*Significant*
◦ Distance to health facility	1	2,164	1.01 (1.00–1.01)	0			*Marginal*
◦ Ownership of house *(ref = owner)*	0			1	1,350	1.90 (1.30–2.80)	*Marginal*
◦ Severe malaria	0			1	1,350	3.60 (2.20–5.90)	*Significant*
**Neglected tropical diseases**							
◦ Residence *(ref = urban)*	1	92	0.56 (0.08–3.33)	0			*Marginal*
◦ Socioeconomic status *(ref = wealthiest)*	2	203	3.98 (0.76–20.93)	0			*Significant*
◦ Insurance status *(ref = insured)*	1	111	2.13 (0.10–44.80)	0			*Marginal*
◦ Sex of the patient *(ref = male)*	1	92	0.67 (0.28–1.67)	0			*Marginal*
◦ Age of the patient	1	92	1.20 (0.30–4.40)	0			*Marginal*
◦ Education status of the patient	1	92	0.67 (0.12–1.25)	0			*Marginal*
◦ Religion of the patient *(ref = Christian)*	1	92	2.60 (0.40–15.9)	0			*Marginal*

Abbreviations: **UVD**: Uncomplicated vaginal delivery. **NS**: Not significant.

**Table 5:** Study characteristics and main findings of included qualitative studies^a^ (n = 7).

Study *Country*	Qualitative methods		Participants sample size	Study objectives	Main findings
	Data collection	Data Analysis			
Botman et al. 2021 *Tanzania*	Observation, discussion groups, and unstructured interviews	Grounded theory	67	To investigate patients’ access to surgical care for burns in terms of timeliness, surgical capacity, and affordability in a regional referral hospital in Manyara, Tanzania	* Hospitalization induced CHE incidence, exceeding CHE threshold by up to 6 times for contracture patients and up to 15 times for acute burn wounds patients. * Despite accepting hospital fees in instalments, patients faced debts that became large burden for the families involved. * Common coping mechanism was selling land and animals, assets, as well as rely on neighbours to feed their children.
Chabrol et al. 2019 *Cameroun*	Individual in-depth interviews	Grounded theory	12	To appraise patients’ and healthcare professionals’ (HCP) experiences with hepatitis B virus (HBV) and hepatitis C virus (HCV) diagnosis and care, with respect to diagnosis, counselling, access to care and treatment, and the infections’ impacts on social and economic trajectories of patients in Yaoundé, Cameroun	* Access to care and treatment for HBV and HCV infection depends on patients’ capacity to pay for these expensive tests * For HBV and HCV patients who do pay for these screenings, the consequences on their social and economic life trajectories are catastrophic. * Patients with HIV-HBV co-infection experienced less barriers to accessing treatments as their HIV antiretroviral treatment (tenofovir) was also effective for HBV. Others experienced OOP payments that were insurmountable barriers to access care. * The OOP expenditures required for treatment impacted detrimental financial consequences including debts, selling assets, and relying on financial support of social network.
Ibukun & Adebayo 2020 *Nigeria*	Individual in-depth interviews	Discourse analysis	27	To assess the level of poverty among those with NCDs, the OOP expenses incurred on NCDs while considering the probability of NCDs inducing CHE and impoverishment	* NCDs induce CHE and leads to impoverishment particularly for households in the lowest socio-economic quintile. * Health insurance reduced the probability of CHE incidence from NCDs healthcare.
Ntambue et al. 2019 *Congo*, *Democratic Republic*	Semi-structured individual interviews	Content analysis	58	To identify risk factors for CHE incidence associated with obstetric and neonatal care in Lubumbashi, Congo DR.	* Hospitalization cost for obstetric and neonatal care–unknown at admission–were a great burden which the household struggle with. * CHE incidence was higher among poor households, maternal or neonatal complications, and involved specialist care * Inability to meet hospitalization costs lead to incarceration of mothers and newborn, and impoverishment
Nwanna-Nzewuna et al. 2021 *Uganda*	Semi-structured individual interviews	Grounded theory	546	To determine the societal cost of surgical care delivery and its drivers; to ascertain the prevalence of CHE incidence and medical impoverishment among surgical patients at Soroti Regional Referral Hospital (SRRH), Uganda and their households; and to elucidate the impact of surgical hospitalization on patients and their households	* Hospitalization induced severe financial catastrophe and impoverishment for the households * Lost income-earning opportunities complicates family finances during surgical hospitalization.
Obembe & Fonn 2020 *Nigeria*	Individual in-depth interviews	Inductive (reasoning) analysis	31	To explore the lived experiences of people admitted for a recent emergency surgical procedure in selected hospitals in Ibadan, Nigeria with a specific focus on both slum and non-slum dwellers	* Health insurance coverage and social health insurance participation was very low * CHE incidence and inability to pay leads to delayed or poor-quality care, humiliation, and incarceration * CHE incidence and inability to pay is worse among low-income households
Onarheim et al. 2018 *Ethiopia*	Individual in-depth interviews (IDI) and focus-group discussions (FGD)	Content analysis	41 IDIs and 7 FGDs	To explore intra-household resource allocation, focusing on how families prioritize newborn health and household needs in Ethiopia; and to explore coping strategies families use to manage these priorities.	* Even though child and maternal health services are supposed to be provided free of charge at the health center level, families still suffer CHE incidence for newborn care at the hospital. * Families are forced to choose between potential worsening of the baby’s health on the one hand, and risking unbearable newborn healthcare costs or financial consequences for the family when taking the newborn to hospital * The poorest households are most faced by CHE incidence from newborn care, with little or no coping mechanism

^a^Only findings from qualitative analysis were reported here. Quantitative data in mixed methods studies were included meta-analysis and narrative synthesis.

**Table 6:** Themes, subthemes and number of contributing statements and studies with examples of supporting statements from qualitative studies.

Theme	Subtheme	Statements (n)	Studies (n)	Examples of supporting statements with citation number of contributing study
Low socioeconomic status	Poor households faced financial barriers to accessing healthcare	13	5	*“The speed with which patients get care depends on the head of the family’s pocket”* [36] *“The problem we have with hepatitis B is the exorbitant cost of the assessment for patients*, *who are most often students and cannot afford to pay*, *so we can’t follow them*.*”* [36] *“The drugs I am asked to buy cost between 6000 and 7000 (about $20 and $24)*. *Just drugs*! *Sometimes it may not even last me for the whole month*. *As a pensioner who retired from a state where our pension has not been paid for so long*, *it is serious*. *Even though it is not supposed to be big money if the economy was good and things are normal*, *but in truth*, *this is where we have found ourselves”* [51] ^a^ *“Since I had no money to go to the health center*, *when my daughter fell ill*, *I went to get*, *on credit*, *malaria medications from the pharmacy of my friend’s little brother*.*”* [77] *“*…*Yes*, *I was delayed because of money problem so I was a bit delayed”* [81] *“If I go to the hospital with my child*, *there is no one who can properly give food for the others*, *there is no one to wash them or send them to school properly*. *They will not go to school and also there will be no one to buy them books*.*”* [87]
	Poor households that access care face financial catastrophe	11	5	*“We sold our land (USD 805) to access treatment”* [79]. *“Another said*, *“We sold food stuff (USD 54)*, *2 goats (USD 97)*, *a bull (USD 258)*, *a pig (USD 43)”* [79] *“I had asked the nurses to keep my baby if they wanted*, *and to let me go look for money until I could pull together the necessary sum*.*”* [77]
	Lost income-earning opportunities complicates access to care	8	3	*“Since I was in the hospital*, *I couldn’t trade*, *and I couldn’t help my husband*: *everything was screwed [messed up]*. *On his own*, *he had to pay for everything*: *food*, *school fees*, *transportation*, *clothes*, *etc*. *In these conditions*, *that’s how we didn’t have money to pay for health care*.*”* [77] *“Let us say a person has an ox with which he farms his land*. *If he sells this ox to be able to pay for treatment for his child*, *he will have nothing to fend his family with*. *…”* [87] “*I went crying to my older sisters*. *They gave me money to open a small business*. *I spent everything to pay for the exams*. *Now I am here with no money (…)”* [36]
Not having health insurance	Health insurance coverage is low	7	3	*“Only a few people benefit from insurance schemes—like civil servants*, *or people whose employers have an insurance scheme—and have access to this programme (for pre-therapeutic assessment)*, *but they still have to pay for the injections*.*”* [36] *“Quite a number of people here are not extremely poor because healthcare in Nigeria is not cheap*. *The extreme poor will not come to the hospital because insurance is minimal*, *although some may come when it is life-threatening*.*”* [51].
	Health insurance enrolment should be encouraged	5	3	*“Health insurance makes a lot of things cheap for me*. *I collect the drugs at almost no cost*, *even when I pay 1000*, *it doesn’t even matter because I know the drugs that I am given cost much more than that*. *The other day*, *they didn’t have the drugs I wanted*, *when I got to a pharmacy outside and I bought it with my money*, *then I realized I how much I have been enjoying*.*”* [51] *“It is supposed to be available for everybody*, *not government workers alone*. *We are all Nigerians*, *so NHIS should be available for everybody”* [81]

^a^Currency converted to US Dollars using the prevailing exchange rate at the time of study

### Population-level factors

#### Household characteristics

Household characteristics that are associated with CHE incidence include residence [16, 20, 21, 28, 39, 41, 43, 45, 46, 50, 52, 56, 58, 62–65, 67, 68, 73–76, 78, 91–94, 98, 99, 103], socioeconomic status [16, 17, 21, 25–28, 34, 35, 39, 41, 43, 45, 46, 50, 52, 53, 55, 56, 58, 59, 62, 63, 65–68, 73, 75, 83, 91–93, 95, 98, 99, 103, 104], household size [17, 20, 21, 25, 26, 28, 34, 39, 41, 43, 45, 50, 53, 58, 62, 63, 65, 66, 68, 73, 75, 76, 83, 92, 93, 95, 98, 99, 104], health insurance status [17, 18, 21, 23, 26–28, 39, 44, 46, 47, 52, 53, 59, 62, 64, 66, 74–76, 83, 92, 94], social safety recipient [27, 35, 38], and marginalization status [28]. Meta-analysis of comparable studies suggests that only socio-economic status (10% THE: OR = 1.99 (95% CI = 1.32–2.98) and 40% NFE: OR = 3.02 (95% CI = 2.23–4.08)) and household size (10% THE: OR = 1.07 (95% CI = 1.02–1.13) and 40% NFE: OR = 1.06 (95% CI = 1.00–1.12)) were significantly associated with CHE incidence (**Table 3**).

Rural households are at a particularly high risk of catastrophic costs. A multi-country World Health Survey showed that “households living in urban areas consistently seemed to be better protected against catastrophic health expenditure” than rural households [91]. Rural residence, combined with distance to health facilities, increases rural households’ exposure to financial catastrophe[52].

The poorest households were at a higher risk of CHE than richer households [28, 43, 46, 51, 53, 81, 87, 91], as the following statement from a respondent reflects:

*“I got treatment for my first child from the hospital*, *and they charged us a lot of money*. *We did not have anything left after*, *and my husband was hiding*. *After a long time*, *we were able to borrow money from a relative…”* [87]

Health insurance coverage and social safety nets both protect households from CHE, although quantitative analysis suggests this protection is inconsistent.

*“Health insurance makes a lot of things cheap for me*. *I collect the drugs at almost no cost*, *even when I pay 1000*, *it doesn’t even matter because I know the drugs that I am given cost much more than that*. *The other day*, *they didn’t have the drugs I wanted*, *when I got to a pharmacy outside and I bought it with my money*, *then I realized I how much I have been enjoying”* [51].

#### Household head factors

Several studies reported the relationship between CHE incidence and the sex/gender [17, 21, 28, 34, 35, 39, 41, 43, 45, 50, 52, 55, 56, 58, 62, 63, 65–68, 73, 75, 76, 78, 91–93, 95, 98, 99, 103, 104], age [17, 25, 28, 34, 35, 39, 43, 46, 50, 52, 58, 65–68, 75, 76, 92, 95, 104], marital status [20, 39, 43, 45, 62, 63, 76, 78, 92, 93, 95, 98, 104], education status [17, 20, 21, 26, 34, 39, 43, 45, 50, 56, 62, 63, 65–67, 73, 75, 76, 78, 91–93, 95, 99, 103, 104], and employment status [17, 21, 28, 35, 39, 43, 45, 46, 50, 52, 56, 62, 63, 67, 73, 75, 76, 78, 92, 95, 104] of the household head. Of these factors, only the employment status was significantly associated with CHE incidence (**Table 3**). In settings without universal insurance coverage, when the household head (who are often the main, or even the only, income earner) is unable to work due to own or a family member’s illness, the combination of lost income and health expenses is devastating [81, 87]. Also, households headed by a retiree were particularly at high risk of CHE incidence, as high as 75% [28, 78].

#### Household members factors

CHE incidence was significantly associated with advanced age [17, 21, 26, 28, 39, 41, 43, 45, 50, 52, 56, 62, 63, 65, 68, 73, 75, 78, 83, 91–93, 98, 103], chronic illness [21, 25, 26, 28, 34, 39, 45, 56, 62, 67, 68, 74–76, 83, 92, 93, 95, 99], and hospitalization [17, 25, 27, 41, 52, 58, 62, 68, 83, 94, 103, 104]; but not associated with presence of children < 5-years of age [21, 35, 39, 41, 43, 45, 50, 62, 63, 65, 66, 68, 73, 75, 83, 91, 98], women of child-bearing age [50], disability [17, 34, 65, 66, 91, 98, 99], or obesity [39] in the household (**Table 3**). Tobacco smoking increased the likelihood of CHE incidence (OR = 1.11 (95% CI = 1.10–1.12)) [16].

#### Health system factors

Several studies evaluated the link between CHE incidence and the level of health facility were care was sought [25, 35, 45, 56, 67], health facility type [17, 21, 25, 35, 39, 45, 52, 56, 68, 73, 75, 93, 94], distance to health facility [41, 46, 56, 58, 62, 65, 67, 68, 93], number of health facilities in district/county [28], and prior care from traditional healers [27, 34]. Of these, health facility type, and health facility level were significantly associated with CHE incidence–**Table 3**. A few studies, however, showed that accessing care from private healthcare providers decreased households’ risk of catastrophic expenditure, although the level and type of care sought from these providers was not clear [21, 52, 93].

#### Other factors

Other marginal factors linked with CHE incidence at the population level include violence against women [34], house ownership [46], business ownership [35], and regular use of mosquito bed nets [17, 52]–**Table 3**.

### Disease-specific determinants

#### Non-communicable diseases (NCDs)

NCDs significantly increased households’ likelihood of incurring CHE. Cancer increased the likelihood of a household incurring CHE by 7.6%, diabetes 3.5%, TB 3.4%, hypertension 1.9%, and other cardiac diseases by 0.9%. Overall, having a chronic diseases member in a household increased the likelihood of CHE incidence by 2.2% [80]. For households affected by NCDs, CHE incidence was significantly associated with poor socioeconomic status [48, 49, 51, 57, 80, 96, 100, 101], employment status [51, 57, 80, 96, 100, 101], old age [48, 49, 51, 80, 96], and disability [48]. However, household head’s sex [48, 49, 51, 57, 71, 80, 101], marital status [51, 57, 71, 80, 96, 101], education status [48, 49, 51, 57, 71, 80, 101], employment status [51, 57, 80, 96, 100, 101], household residence [48, 49, 57, 71, 80, 100, 101], and religion [101] were not associated with CHE incidence (**Table 4**). Having health insurance was protective of catastrophic costs [51, 71, 80]–as in the population level.

#### Reproductive, neonatal, and child healthcare

For households that sought reproductive, newborn, and child healthcare, CHE incidence was linked to household residence [22], socioeconomic status [38, 42, 77], household size [42], health insurance [42], education status [22, 30, 42], employment status [38, 42], health facility level [77], type of healthcare provider [77], distance to health facility [22], pre-natal illness/hospitalization [77], complicated delivery [77], HIV+ pregnancy [42], and neonatal admission [77]–**Table 4**. Of these, household residence, socio-economic status, insurance status, household head employment status, pre-natal hospitalization, delivery complications, and neonatal admission were significantly associated with CHE incidence.

“*I had asked the nurses to keep my baby if they wanted*, *and to let me go look for money until I could pull together the necessary sum*.” [77]

*“I got treatment for my first child from the hospital*, *and they charged us a lot of money*. *We did not have anything left after*, *and my husband was hiding*. *After a long time*, *we were able to borrow money from a relative…”* [87]

#### Surgery and trauma care

For households that sought surgical or trauma care, CHE incidence was associated with residence, socioeconomic status, health insurance status, and sex, age, marital status, education, and employment status of household head–**Table 4**. Other factors include old age, hospitalization, healthcare provider type, specialist care, intensive care unit admission, and emergency surgery [31, 40, 79, 81, 82, 84, 89, 90].

*“*…*all my family ran away because of the [surgical] expenses*‥*”* [81]

#### Chronic infectious disease (HIV, TB, HBV, and HCV)

CHE incidence for households that sought healthcare for HIV, TB, HBV, and HCV infections was linked to 19 sociodemographic and health system factors [24, 29, 32, 33, 36, 38, 61, 69, 72, 80, 102] (**Table 4**). Of these, socioeconomic status [24, 29, 38, 69, 72, 80, 102], health insurance [24, 29, 72, 80], employment status [29, 36, 38], hospitalization [24, 102], healthcare provider type [24, 102], HIV-TB coinfection [24, 69, 102], and extra-pulmonary TB [24] were significantly associated with CHE incidence. Notably, while HIV care decentralization improves equity in access to ART, it does not fully remove the risk of CHE, unless other innovative reforms in health financing are implemented [33]. While HIV patients’ healthcare is largely subsidized, the costs of TB, HBV, HCV care are mostly borne directly by the patients. Therefore, the latter households face significantly higher risks of CHE [36, 61, 80].

#### Malaria

The included studies identified six sociodemographic factors—household residence, socioeconomic status, household head’s sex, age, education, and employment status—and two health system factors: healthcare provider type and distance to the health facility [54, 97]. Of these, only socioeconomic status was significantly associated with CHE incidence for malaria treatment (**Table 4**).

#### Neglected tropical diseases (NTDs)

For households that sought healthcare for NTDs, seven socio-demographic factors—household residence, socio-economic status, health insurance, and the sex, age, education, and religion of the patients—were linked with CHE incidence [37, 85] (**Table 4**). Of these factors, only socioeconomic status was significantly associated with CHE incidence.

## Discussion

Factors associated with CHE incidence among SSA households are multidimensional and diverse. Overall, a few points emerge from this review. First, the majority of included studies used regression analysis to evaluate the factors associated with CHE incidence. Given that included studies utilized different definitions for evaluated factors, meta-analysis was possible for fewer included studies. However, all included studies were evaluated and synthesized narratively. Secondly, studies evaluating CHE incidence in SSA countries mostly used the ‘capacity-to-pay’ or ‘non-food expenditure’ definition while fewer studies used the ratio of OOP to total household income [7]. However, studies that used both definitions suggests that CHE-associated factors were largely similar between the definitions [19, 21, 30, 60, 68, 78, 92, 93]. Reporting CHE incidence and CHE-associated factors using both definitions enhances comparability between studies. Also, despite the progress SSA countries have made towards universal health insurance, households are still exposed to CHE [46, 66, 84]. Yet, it is likely that many low-income uninured households in SSA countries without universal insurance choose not to seek health care rather than face the financial hardship associated with out-of-pocket healthcare payments [46, 51, 99].

At the population level, our review highlights rural residence, low socioeconomic status, lack of health insurance, advanced age, chronic illness, hospitalization, utilization of private healthcare provider, and utilization of specialist care as the most significant determinants of CHE incidence. Our findings are consistent with findings in comparable regions such as Southeast Asia [105, 106] and South America [107, 108]. Due to widespread poverty, most SSA households cannot afford insurance premiums and so rely on OOP payment for healthcare [2, 109]. Given the highly regressive impact of OOP payment [2, 3], most studies in SSA region demonstrate households’ socioeconomic status as a risk factor for CHE [3, 109]. Rural residence in SSA countries is a proximal indicator of limited household income [50, 91, 103]. This is compounded by lack of health facilities in the rural settings, transportation costs to reach urban health facilities, or the indirect expenditure, such as the costs incurred by an accompanying caretaker[20, 21, 76, 91]. Having an elderly person in the household increases the chances of incurring CHE [21, 26, 63, 103]. This is as expected because elderly persons require more healthcare [21], and are more likely to have chronic illnesses [26, 28]. Both factors increase health expenditures and often require working family members to quit their jobs. Hospitalization, utilization of private healthcare provider, and/or specialist (tertiary) healthcare all increase the possibility of incurring CHE [25, 41, 62, 75, 94]. Given that most SSA countries do not have financial risk protection mechanisms in place, this situation is even grim as the CHE definitions used in included studies does not consider households with unmet healthcare needs.

Factors distinctly associated with CHE incidence at the disease-specific level include disability in a household member for NCDs; severe malaria, blood transfusion, and distant health facilities for maternal and child health services; emergency/unplanned surgery for surgery and trauma patients; and low CD4 count, HIV and TB co-infection, and extra-pulmonary TB for HIV and TB patients. For households affected by NCDs, disability imposes further financial burden in the form of extra health expenses and lost income [51]. The farther the distance of health facilities from the place of residence, the higher the direct non-medical costs, including transportation and accommodation costs. Hence, rural households are therefore more likely to incur CHE for maternal and child healthcare [22, 97]. For similar reasons, blood transfusion and severe malaria treatments are rarely available at rural health facilities, and require hospitalization and specialist care–which increase CHE risks [22, 54]. For patients requiring HIV and TB care, low CD4-count, HIV and TB co-infection, and extra-pulmonary TB are all indicative of poor health status requiring increased usage of healthcare services with a higher risk of incurring CHE [24, 29, 102].

### Strengths and limitations

To the best of our knowledge, this is the first systematic review to comprehensively map the factors associated with CHE incidence in SSA. We also identified determinants for both population and disease-specific level CHE incidence which enables easy identification of populations that are most at risk for community-wide and/or vertical disease-specific interventions. Furthermore, our review combined both quantitative and qualitative studies to synthesize evidence that is both generalizable and sufficiently nuanced.

Our study has a few limitations. First, our review does not capture factors associated with households who cannot meet treatment costs–a gap that future studies can address using new variables that capture these households. Also, as we identified determinants of CHE incidence using two thresholds, we may have missed some factors that might have been reported using other thresholds. Thirdly, there is the inherent difficulty in mapping and adjudicating the evidence on these factors identified from the studies as either significant or marginal. Ultimately, these were subjective judgments based on the authors’ understanding of the texts in included studies that are not as error-proof as might be hoped for. To address this, a multi-rater system was used–each factor was independently adjudicated by at least two authors–to minimize subjectivity. Finally, our categorization of some determinants as marginal does not imply dismissal of the influence of these factors in some unique settings. In some settings and for different households, these “marginal” factors could have greater eminence.

### Policy implications

Our review provides significant contextual evidence for policy discussion and health financing reforms by identifying the sociodemographic characteristics of households that are most likely to suffer financial catastrophe in SSA countries. This is a critical step toward developing comprehensive social protection mechanisms–a key vehicle for achieving UHC. Our study provides key details for fine-tuning the different means of identifying households for targeted or supplemental protection such as means testing, proximal means testing, geographic targeting, or participatory wealth ranking [109].

## Conclusion

Our study suggests that the key factors associated with population and disease-specific CHE incidence in SSA countries are rural residence, low socioeconomic status, lack of health insurance, having an elderly household member, chronic illness, hospitalization, use of private healthcare providers, and use of tertiary/specialist healthcare. Highlighting these factors in a comprehensive review underscores potential strategies for implementing/improving financial risk protection measures to achieve UHC in these SSA countries.

## Supporting information

S1 Table Search strategy. Search period was from 01 January 1990 to 31 December 2021.
(DOCX)

S2 Table Eligibility criteria for studies reporting factors associated with catastrophic health expenditure in sub-Saharan Africa (SSA) countries. (DOCX)

S1 Fig Thematic analysis map. (TIF)

S1 Checklist PRISMA 2009 checklist. (DOC)
