# Supplementary material for: Factors associated with catastrophic health expenditure in sub-Saharan Africa: A systematic review
Source: PLoS One. 2022 Oct 20;17(10):e0276266. doi: 10.1371/journal.pone.0276266 (PMC9584403; doi:10.1371/journal.pone.0276266)
Supplement: S1 Table — Search period was from 01 January 1990 to 31 December 2021. (DOCX) [file pone.0276266.s001.docx]

**Supplement 1:** Search strategy

| **DATABASES** | **Results*** |
| --- | --- |
| **PubMed / MEDLINE** | 286 |
| Search: ((("risk"[MeSH Terms] OR "risk"[All Fields]) AND "factor*"[All Fields]) OR "determi*"[All Fields] OR (("health"[MeSH Terms] OR "health"[All Fields] OR "health s"[All Fields] OR "healthful"[All Fields] OR "healthfulness"[All Fields] OR "healths"[All Fields]) AND ("risk"[MeSH Terms] OR "risk"[All Fields]) AND ("family"[MeSH Terms] OR "family"[All Fields] OR "relation"[All Fields] OR "relatability"[All Fields] OR "relatable"[All Fields] OR "related"[All Fields] OR "relates"[All Fields] OR "relating"[All Fields] OR "relational"[All Fields] OR "relations"[All Fields])) OR (("factor"[All Fields] OR "factor s"[All Fields] OR "factors"[All Fields]) AND ("associate"[All Fields] OR "associated"[All Fields] OR "associates"[All Fields] OR "associating"[All Fields] OR "association"[MeSH Terms] OR "association"[All Fields] OR "associations"[All Fields]))) AND ((("catastrophe"[All Fields] OR "catastrophes"[All Fields] OR "catastrophic"[All Fields] OR "catastrophically"[All Fields]) AND ("health"[MeSH Terms] OR "health"[All Fields] OR "health s"[All Fields] OR "healthful"[All Fields] OR "healthfulness"[All Fields] OR "healths"[All Fields]) AND "expen*"[All Fields]) OR (("catastrophe"[All Fields] OR "catastrophes"[All Fields] OR "catastrophic"[All Fields] OR "catastrophically"[All Fields]) AND ("health"[MeSH Terms] OR "health"[All Fields] OR "health s"[All Fields] OR "healthful"[All Fields] OR "healthfulness"[All Fields] OR "healths"[All Fields]) AND "cost*"[All Fields]) OR (("catastrophe"[All Fields] OR "catastrophes"[All Fields] OR "catastrophic"[All Fields] OR "catastrophically"[All Fields]) AND ("health"[MeSH Terms] OR "health"[All Fields] OR "health s"[All Fields] OR "healthful"[All Fields] OR "healthfulness"[All Fields] OR "healths"[All Fields]) AND "payment*"[All Fields]) OR (("catastrophe"[All Fields] OR "catastrophes"[All Fields] OR "catastrophic"[All Fields] OR "catastrophically"[All Fields]) AND "out-of-pocket"[All Fields] AND "payment*"[All Fields]) OR (("economics"[MeSH Terms] OR "economics"[All Fields] OR "financial"[All Fields] OR "financially"[All Fields] OR "financials"[All Fields] OR "financier"[All Fields] OR "financiers"[All Fields]) AND ("catastrophe"[All Fields] OR "catastrophes"[All Fields] OR "catastrophic"[All Fields] OR "catastrophically"[All Fields]))) AND (("Sub-Sahara"[All Fields] AND ("africa"[MeSH Terms] OR "africa"[All Fields] OR "africa s"[All Fields] OR "africas"[All Fields])) OR ("africa south of the sahara"[MeSH Terms] OR ("africa"[All Fields] AND "south"[All Fields] AND "sahara"[All Fields]) OR "africa south of the sahara"[All Fields] OR ("sub"[All Fields] AND "saharan"[All Fields] AND "africa"[All Fields]) OR "sub saharan africa"[All Fields]) OR ("Sub-Saharan"[All Fields] AND ("africans"[All Fields] OR "blacks"[MeSH Terms] OR "blacks"[All Fields] OR "african"[All Fields])) OR ("angola"[MeSH Terms] OR "angola"[All Fields] OR "angola s"[All Fields] OR ("benin"[MeSH Terms] OR "benin"[All Fields] OR "benin s"[All Fields]) OR ("botswana"[MeSH Terms] OR "botswana"[All Fields] OR "botswana s"[All Fields]) OR ("burkina faso"[MeSH Terms] OR ("burkina"[All Fields] AND "faso"[All Fields]) OR "burkina faso"[All Fields]) OR ("burundi"[MeSH Terms] OR "burundi"[All Fields]) OR ("cabo verde"[MeSH Terms] OR ("cabo"[All Fields] AND "verde"[All Fields]) OR "cabo verde"[All Fields]) OR ("cameroon"[MeSH Terms] OR "cameroon"[All Fields] OR "cameroons"[All Fields] OR "cameroon s"[All Fields]) OR ("central african republic"[MeSH Terms] OR ("central"[All Fields] AND "african"[All Fields] AND "republic"[All Fields]) OR "central african republic"[All Fields]) OR ("chad"[MeSH Terms] OR "chad"[All Fields]) OR ("comoros"[MeSH Terms] OR "comoros"[All Fields] OR "comoro"[All Fields]) OR "democratic republic congo"[All Fields] OR "republic congo"[All Fields] OR "Cote d'Ivoire"[All Fields] OR ("equatorial guinea"[MeSH Terms] OR ("equatorial"[All Fields] AND "guinea"[All Fields]) OR "equatorial guinea"[All Fields]) OR ("eritrea"[MeSH Terms] OR "eritrea"[All Fields]) OR ("eswatini"[MeSH Terms] OR "eswatini"[All Fields]) OR ("ethiopia"[MeSH Terms] OR "ethiopia"[All Fields] OR "ethiopia s"[All Fields]) OR ("gabon"[MeSH Terms] OR "gabon"[All Fields]) OR ("gambia"[MeSH Terms] OR "gambia"[All Fields] OR "the gambia"[All Fields]) OR ("ghana"[MeSH Terms] OR "ghana"[All Fields] OR "ghana s"[All Fields]) OR ("guinea"[MeSH Terms] OR "guinea"[All Fields] OR "guinea s"[All Fields] OR "guineas"[All Fields]) OR ("guinea bissau"[MeSH Terms] OR "guinea bissau"[All Fields] OR ("guinea"[All Fields] AND "bissau"[All Fields]) OR "guinea bissau"[All Fields]) OR ("kenya"[MeSH Terms] OR "kenya"[All Fields] OR "kenya s"[All Fields]) OR ("lesotho"[MeSH Terms] OR "lesotho"[All Fields]) OR ("liberia"[MeSH Terms] OR "liberia"[All Fields] OR "liberia s"[All Fields]) OR ("madagascar"[MeSH Terms] OR "madagascar"[All Fields] OR "madagascar s"[All Fields]) OR ("malawi"[MeSH Terms] OR "malawi"[All Fields] OR "malawi s"[All Fields]) OR ("mali"[MeSH Terms] OR "mali"[All Fields]) OR ("mauritania"[MeSH Terms] OR "mauritania"[All Fields]) OR ("mauritius"[MeSH Terms] OR "mauritius"[All Fields]) OR ("mozambique"[MeSH Terms] OR "mozambique"[All Fields] OR "mozambique s"[All Fields]) OR ("namibia"[MeSH Terms] OR "namibia"[All Fields]) OR ("niger"[MeSH Terms] OR "niger"[All Fields]) OR ("nigeria"[MeSH Terms] OR "nigeria"[All Fields] OR "nigeria s"[All Fields]) OR ("rwanda"[MeSH Terms] OR "rwanda"[All Fields] OR "rwanda s"[All Fields]) OR "Sao Tome and Principe"[All Fields] OR ("senegal"[MeSH Terms] OR "senegal"[All Fields] OR "senegal s"[All Fields]) OR ("seychelles"[MeSH Terms] OR "seychelles"[All Fields]) OR "Sierra Leone"[All Fields] OR ("somalia"[MeSH Terms] OR "somalia"[All Fields]) OR "South Africa"[All Fields] OR "South Sudan"[All Fields] OR ("sudan"[MeSH Terms] OR "sudan"[All Fields] OR "sudans"[All Fields] OR "sudan s"[All Fields]) OR ("tanzania"[MeSH Terms] OR "tanzania"[All Fields] OR "tanzania s"[All Fields]) OR ("togo"[MeSH Terms] OR "togo"[All Fields]) OR ("uganda"[MeSH Terms] OR "uganda"[All Fields] OR "uganda s"[All Fields]) OR ("zambia"[MeSH Terms] OR "zambia"[All Fields] OR "zambia s"[All Fields]) OR ("zimbabwe"[MeSH Terms] OR "zimbabwe"[All Fields] OR "zimbabwe s"[All Fields]))) |  |
|  |  |
| **Scopus** | 311 |
| TITLE-ABS-KEY ( catastrophic AND health AND expenditure ) AND ( LIMIT-TO ( AFFILCOUNTRY , "Nigeria" ) OR LIMIT-TO ( AFFILCOUNTRY , "South Africa" ) OR LIMIT-TO ( AFFILCOUNTRY , "Ethiopia" ) OR LIMIT-TO ( AFFILCOUNTRY , "Kenya" ) OR LIMIT-TO ( AFFILCOUNTRY , "Ghana" ) OR LIMIT-TO ( AFFILCOUNTRY , "Uganda" ) OR LIMIT-TO ( AFFILCOUNTRY , "Malawi" ) OR LIMIT-TO ( AFFILCOUNTRY , "Burkina Faso" ) OR LIMIT-TO ( AFFILCOUNTRY , "Tanzania" ) OR LIMIT-TO ( AFFILCOUNTRY , "Sierra Leone" ) OR LIMIT-TO ( AFFILCOUNTRY , "Cameroon" ) OR LIMIT-TO ( AFFILCOUNTRY , "Congo" ) OR LIMIT-TO ( AFFILCOUNTRY , "Zambia" ) OR LIMIT-TO ( AFFILCOUNTRY , "Botswana" ) OR LIMIT-TO ( AFFILCOUNTRY , "Rwanda" ) OR LIMIT-TO ( AFFILCOUNTRY , "Benin" ) OR LIMIT-TO ( AFFILCOUNTRY , "Sudan" ) OR LIMIT-TO ( AFFILCOUNTRY , "Zimbabwe" ) OR LIMIT-TO ( AFFILCOUNTRY , "Madagascar" ) OR LIMIT-TO ( AFFILCOUNTRY , "Somalia" ) OR LIMIT-TO ( AFFILCOUNTRY , "Cote d'Ivoire" ) OR LIMIT-TO ( AFFILCOUNTRY , "Togo" ) OR LIMIT-TO ( AFFILCOUNTRY , "Democratic Republic Congo" ) OR LIMIT-TO ( AFFILCOUNTRY , "Gabon" ) OR LIMIT-TO ( AFFILCOUNTRY , "Gambia" ) OR LIMIT-TO ( AFFILCOUNTRY , "Mali" ) OR LIMIT-TO ( AFFILCOUNTRY , "Mauritania" ) OR LIMIT-TO ( AFFILCOUNTRY , "Mauritius" ) OR LIMIT-TO ( AFFILCOUNTRY , "Mozambique" ) OR LIMIT-TO ( AFFILCOUNTRY , "Namibia" ) OR LIMIT-TO ( AFFILCOUNTRY , "Niger" ) OR LIMIT-TO ( AFFILCOUNTRY , "Senegal" ) OR LIMIT-TO ( AFFILCOUNTRY , "Undefined" ) ) |  |
|  |  |
| **Other databases** |  |
| - AJOL (African Journals Online) | 12 |
| - CINAHL (Cumulative Index of Nursing and Allied Health Literature) via EBSCOHost | 85 |
| - Cochrane CENTRAL (Wiley) | 8 |
| - CNKI (China National Knowledge Infrastructure) | 236 |
| - PsycINFO via ProQuest | 10 |
| - Web of Science: Science Citation Index Expanded, Social Sciences Citation Index & Arts & Humanities Citation Index | 176 |
|  |  |
| **TOTAL SEARCH RESULT** | **1,124** |

* Search period was from 01 January 1990 to 31 December 2021.
