# Supplementary material for: Factors associated with catastrophic health expenditure in sub-Saharan Africa: A systematic review
Source: PLoS One. 2022 Oct 20;17(10):e0276266. doi: 10.1371/journal.pone.0276266 (PMC9584403; doi:10.1371/journal.pone.0276266)
Supplement: S2 Table — (DOCX) [file pone.0276266.s002.docx]

**S2 Table:** Eligibility criteria for studies reporting factors associated with catastrophic health expenditure in sub-Saharan Africa (SSA) countries

| **Inclusion criteria** |
| --- |
| - Publication reporting factors associated with catastrophic health expenditure (CHE) incidence for individuals of all ages in the community identified through household surveys or health facility-based studies that are representative of the entire community. |
| - Quantitative studies: catastrophic health expenditure is defined as either as 10% of total household expenditure or 40% of household non-food expenditure. |
| - Quantitative studies: Observational or pre-interventional studies using population-based data |
| - Qualitative and mixed-methods studies that explored the factors associated with or determinants of catastrophic health expenditure using qualitative data collection methods and qualitative data synthesis methods |
| - Studies conducted in any of the World Bank-identified sub-Saharan African countries: Angola, Benin, Botswana, Burkina Faso, Burundi, Cabo Verde, Cameroun, Central African Republic, Chad, Comoros, Congo Democratic Republic, Congo Republic, Cote d'Ivoire, Equatorial Guinea, Eritrea, Eswatini (Swaziland), Ethiopia, Gabon, The Gambia, Ghana, Guinea, Guinea-Bissau, Kenya, Lesotho, Liberia, Madagascar, Malawi, Mali, Mauritania, Mauritius, Mozambique, Namibia, Niger, Nigeria, Rwanda, Sao Tome and Principe, Senegal, Seychelles, Sierra Leone, Somalia, South Africa, South Sudan, Sudan, Tanzania, Togo, Uganda, Zambia, and Zimbabwe. |
| - English, French, Arabic, Portuguese, Spanish, and Swahili studies |
| - Published and unpublished (Grey) literature |
| **Exclusion criteria** |
| - Publication reporting factors associated with CHE incidence from retrospective patients chart analysis, hospital/pharmacy revenues analysis, or national/subnational budget analysis |
| - Quantitative studies reporting factors for CHE incidence for all individuals of all ages identified in a single or multiple health facility-based studies including outpatient clinic, inpatient hospital, intensive care unit, operating theatres, nursing homes, or long-term care facilities that are not representative of the entire community. |
| - Interventional studies reporting only post-intervention CHE-associated factors. |
| - Factors associated with other catastrophic health expenditure defined using other thresholds or other CHE-related terms such as “Excessive out-of-pocket healthcare expenditure”, multidimensional poverty index (MPI) score, or similar terms |
| - Case reports, case series, reviews, letters to editors, commentary pieces, study protocols |

CHE: Catastrophic health expenditure, HH: Household
