# Supplementary figures and images for: Factors associated with catastrophic health expenditure in sub-Saharan Africa: A systematic review
Source: PLoS One. 2022 Oct 20;17(10):e0276266. doi: 10.1371/journal.pone.0276266 (PMC9584403; doi:10.1371/journal.pone.0276266)

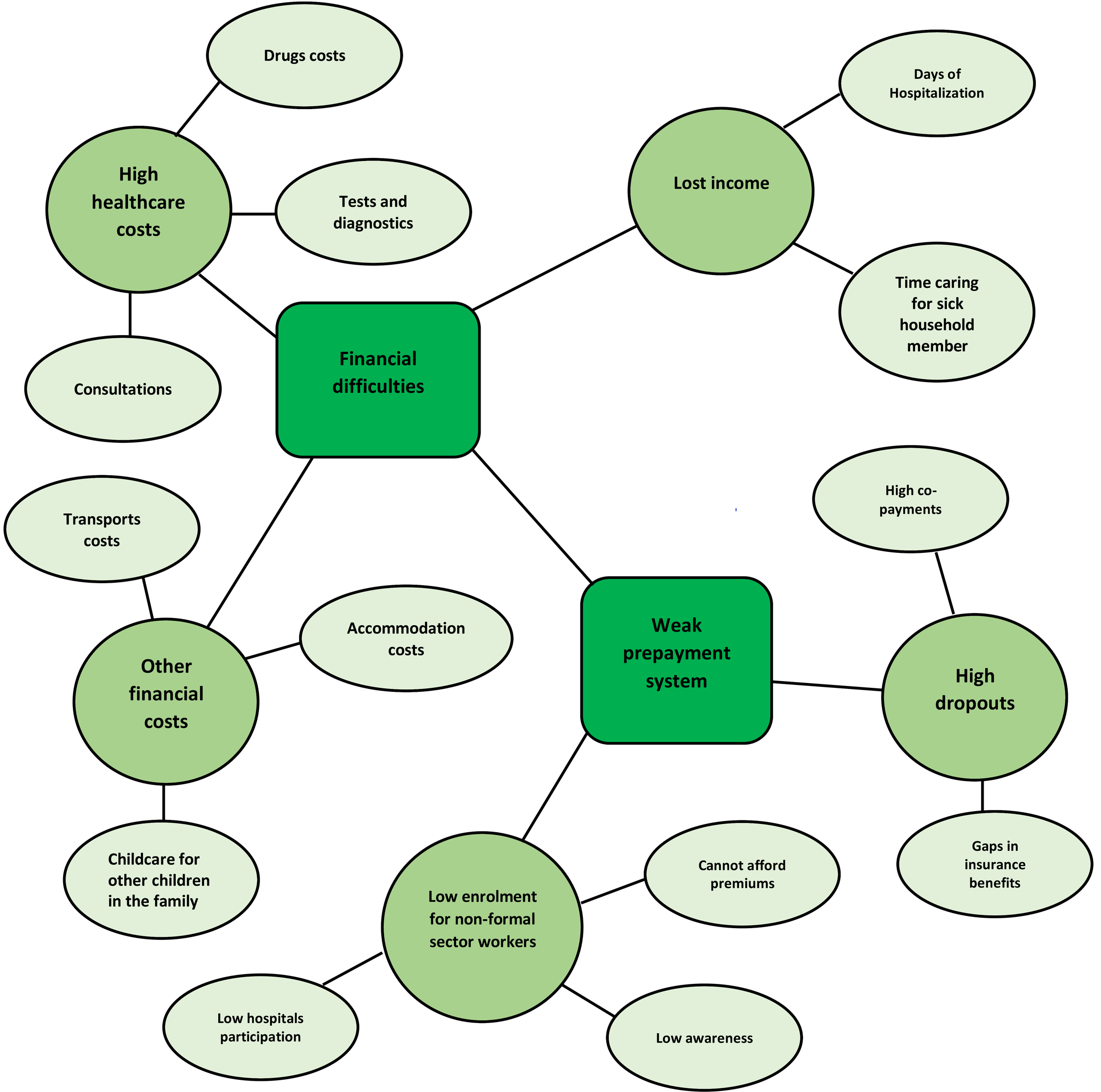

Supplement: S1 Fig — (TIF) [file pone.0276266.s003.tif]
